# Nasal microbiome disruption and recovery after mupirocin treatment in *Staphylococcus aureus* carriers and noncarriers

**DOI:** 10.1038/s41598-022-21453-4

**Published:** 2022-11-17

**Authors:** Valérie O. Baede, Anaïs Barray, Mehri Tavakol, Gérard Lina, Margreet C. Vos, Jean-Philippe Rasigade

**Affiliations:** 1 Department of Medical Microbiology and Infectious Diseases, Erasmus MC University Medical Center Rotterdam, Rotterdam, The Netherlands; 2 CIRI, Centre International de Recherche en Infectiologie, Université de Lyon, Inserm U1111, Ecole Normale Supérieure de Lyon, Université Lyon 1, CNRS, UMR5308, Lyon, France; 3 Centre National de Référence des Staphylocoques, Institut des Agents Infectieux, Hôpital de la Croix Rousse, Hospices Civils de Lyon, Lyon, France

**Keywords:** Medical research, Antimicrobials, Microbial communities, Sequencing, Microbial ecology, Bacterial infection, Metagenomics

## Abstract

Nasal decolonization procedures against the opportunistic pathogen *Staphylococcus aureus* rely on topical antimicrobial drug usage, whose impact on the nasal microbiota is poorly understood. We examined this impact in healthy *S. aureus* carriers and noncarriers. This is a prospective interventional cohort study of 8 *S. aureus* carriers and 8 noncarriers treated with nasal mupirocin and chlorhexidine baths. Sequential nasal swabs were taken over 6 months. *S. aureus* was detected by quantitative culture and genotyped using *spa* typing. RNA-based 16S species-level metabarcoding was used to assess the living microbial diversity. The species *Dolosigranulum pigrum, Moraxella nonliquefaciens* and *Corynebacterium propinquum* correlated negatively with *S. aureus* carriage. Mupirocin treatment effectively eliminated *S. aureus, D. pigrum* and *M. nonliquefaciens,* but not corynebacteria. *S. aureus* recolonization in carriers occurred more rapidly than recolonization by the dominant species in noncarriers (median 3 vs. 6 months, respectively). Most recolonizing *S. aureus* isolates had the same *spa* type as the initial isolate. The impact of mupirocin-chlorhexidine treatment on the nasal microbiota was still detectable after 6 months. *S. aureus* recolonization predated microbiota recovery, emphasizing the strong adaptation of this pathogen to the nasal niche and the transient efficacy of the decolonization procedure.

## Introduction

*Staphylococcus aureus* is an opportunistic pathogen and a frequent cause of severe infections. Approximately 20% of the general population are persistent *S. aureus* carriers and another 30% are intermittent carriers^1^. *S. aureus* is commonly carried in the nose and less frequently in the throat, skin, and perineum^1^.

*Staphylococcus aureus* carriers are at higher risk of infection after invasive procedures and surgery^2,3^. To prevent infections, several countries recommend eliminating *S. aureus* from the nose prior to the at-risk intervention using a decolonization procedure^4^. This typically involves topical antimicrobial treatment with mupirocin nasal ointment with or without chlorhexidine cutaneous body and hair wash. Different decolonization approaches have emerged due to costs and organizational issues in health care^5^. While some advise to treat all patients undergoing at-risk interventions, others limit decolonization to confirmed carriers only.

While we know that the nasal microbiome composition is related to *S. aureus* presence^6,7^, the impact of decolonization procedures on the nasal microbiota is not yet fully understood. In previous nasal microbiome studies, *S. aureus* carriage was associated with higher relative abundances of *Cutibacterium acnes*, *Corynebacterium accolens* and non-aureus staphylococci, and with lower abundances of *Corynebacterium pseudodiphtheriticum*,* Dolosigranulum* spp. and *Cutibacterium granulosum*^6,7^. These associations suggest that the distribution of microbial species in the nose influences *S. aureus* persistence, possibly through competition for nutrients and epithelial binding sites^8^. In turn, the alteration of the microbial distribution after a decolonization procedure might impact the likelihood of persistent *S. aureus* recolonization and, from a clinical standpoint, of decolonization failure. However, the magnitude and duration of microbiota alterations after decolonization are not elucidated. So far, a single-patient microbiome study found shifts in the composition and biodiversity of the nasal microbiota after mupirocin treatment^9^, contrasting with a previous culturomics study of 5 healthy volunteers in which no significant change of microbiota richness and diversity were found up to 1 month after decolonization^10^.

To decipher the relationships between *S. aureus* nasal carriage, the nasal microbiota and decolonization procedures, we conducted a prospective interventional cohort study of *S. aureus* carriers and noncarriers, monitoring microbial community changes over 6 months after mupirocin-chlorhexidine treatment. Using quantitative cultures and 16S metabarcoding, we examined the impact of decolonization on bacterial communities and the delay to recolonization with *S. aureus* and other dominant species.

## Results

### *Staphylococcus aureus* elimination and recolonization

**Figure 1:**
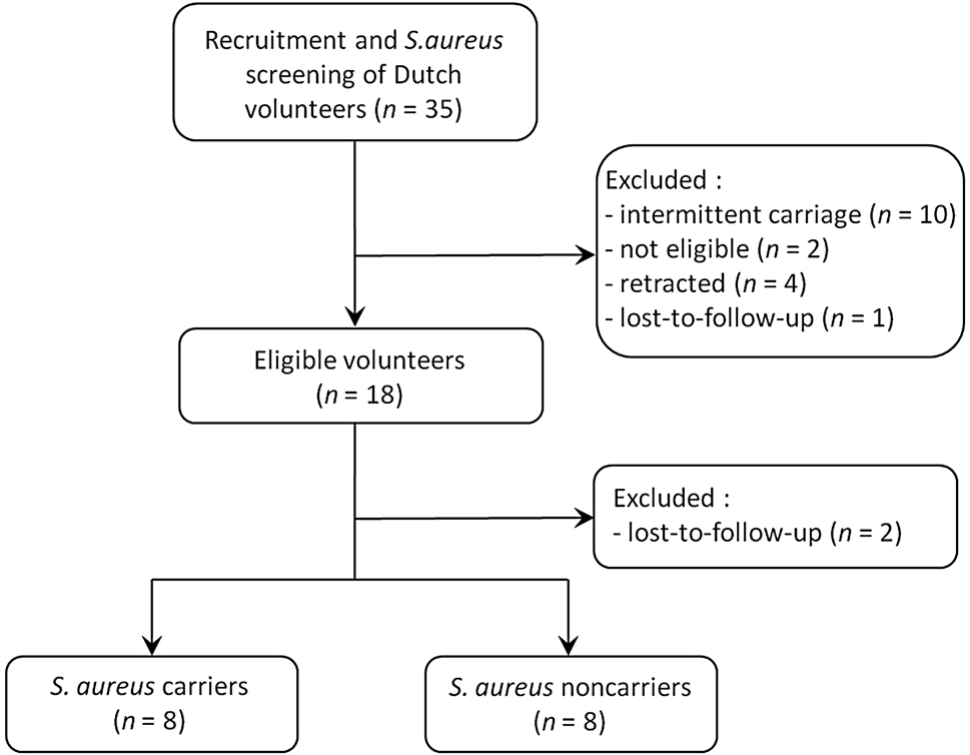
Flowchart of participant recruitment. In total, 35 volunteers were recruited and screened for eligibility. Sixteen participants completed the study, of which 8 carriers and 8 noncarriers.

Of 35 volunteers, 8 carriers and 8 noncarriers were included (see flowchart of patient selection in Fig. 1). The *S. aureus* carrier group consisted of 3 males and 5 females of 22–71 years old (median, 26 years). Noncarriers were 2 males and 6 females aged 18–62 years (median, 56 years). No participants reported the use of antivirals, antiparasitics, immunosuppressants or probiotics in the 3 months prior or during the study. One noncarrier reported the use of amoxicillin/clavulanic acid, 5 days prior to the D0 sampling and again between the M3 and M6 sampling. This participant was retained as antimicrobial use occurred after recruitment and the microbiota composition did not differ from other noncarriers pre-decolonisation. No participants reported previous MRSA carriage. All 16 participants had at least 1 risk factor for *S. aureus* acquisition (Supplementary Table 1).

**Figure 2:**
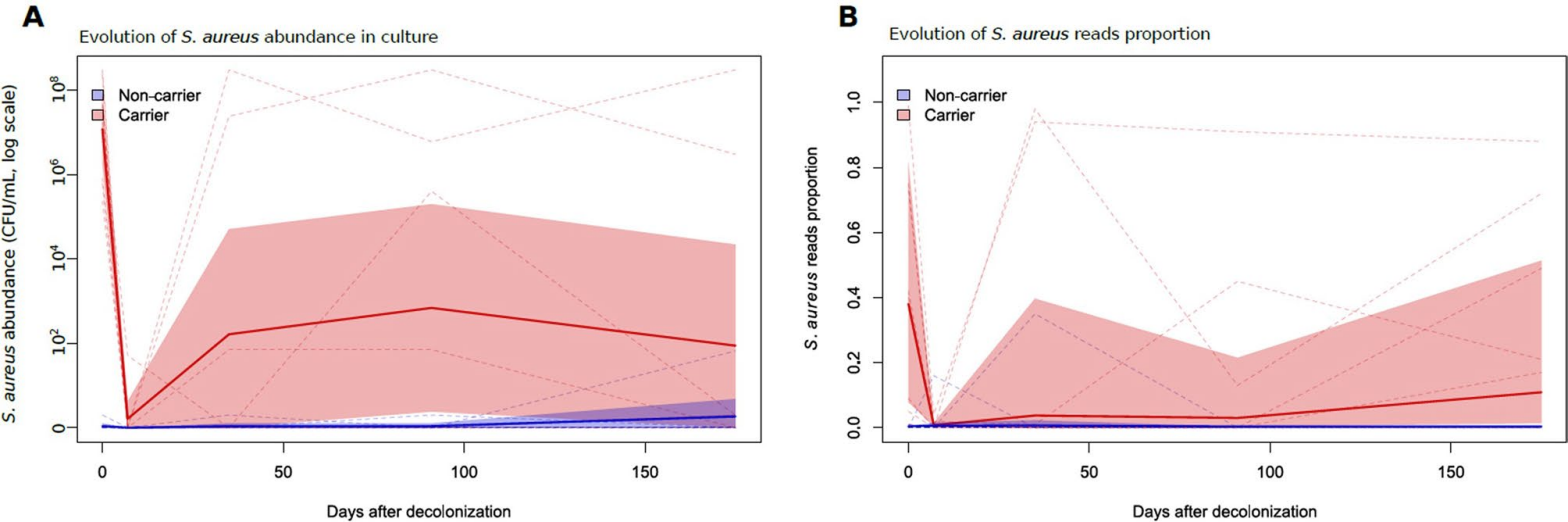
Dynamics of *S. aureus* abundance in nasal samples of carriers and noncarriers undergoing decolonization. Shown are *S. aureus* abundance in quantitative culture (CFU/ml on a log scale; **A**) and proportion in 16S RNA metabarcoding (**B**) through time in 8 carriers (red) and 8 noncarriers (blue). D0 and D7 denote samples taken immediately before and after the decolonization procedure, respectively. Dashed lines denote each participant’s data. Solid lines and colored band denote the mean and 95% confidence interval. Both culture and metabarcoding analysis identified a sharp decrease of *S. aureus* abundance after decolonization followed by recolonization. On average, post-decolonization abundance of *S. aureus* was less than before decolonization.

The dynamics of *S. aureus* elimination and recolonization after the decolonization treatment were examined using quantitative culture (Fig. 2A) and RNA metabarcoding (Fig. 2B). Both methods showed a steep decrease in *S. aureus* loads immediately after decolonization followed by a gradual increase indicating recolonization for some carriers. Failed decolonization in one carrier was confirmed in the first post-decolonization sample by both methods (Fig. 2). Recolonization was defined as a *S. aureus* positive culture (> 8 CFU/mL) post-decolonization. Five carriers (C1, C2, C5, C6 and C7) got recolonized during the follow-up period, including 3 carriers within 1 month post-decolonization. In the noncarrier group, 4 *S. aureus* positive cultures were found post-decolonization, 3 of which with only 1 CFU/mL.

RNA metabarcoding showed different recolonization results. While also 5 carriers (C1, C2, C3, C5 and C8) were recolonized according to RNA metabarcoding, discrepancies were found for 4 carriers (C3, C6, C7 and C8). For 2 carriers (C5 and C8), RNA metabarcoding showed recolonization without a positive culture. Another 2 carriers (C6 and C7) showed no recolonization in RNA metabarcoding despite a positive culture.

*Spa* types were determined in carriers exhibiting *S. aureus* recolonization in culture (n = 5). All but one recolonized *S. aureus* carriers showed the same *spa* type in pre- and post-decolonization samples. In 2 carriers, a different *spa* type was found, suggesting transient colonization by a strain different from the pre-decolonization carriage strain. *Spa* typing results are shown in Table 1. Details of recolonization delay and CFU/mL loads are shown in Supplementary Fig. 1. No phenotypic resistance to methicillin was found in the tested isolates.

**Table 1:** *Staphylococcus aureus spa-*types before and after decolonization in 5 healthy carriers with *S. aureus* recolonization.

Participant	Pre-decolonization *spa*-type	Post-decolonization *spa*-types (delay)
C1	t127	t127 (1 month and 6 months)
C2	t127	t084 (2 days)
C5	t065	t065 (1 and 6 months)
C6	t002	t7568 (3 months), t002 (6 months)
C7	t3884	t3884 (1 and 3 months)

Overall, the *S. aureus* decolonization remained successful over a 6-month period in only 3 participants (38%) (Fig. 2 and Supplementary Fig. 1), consistent with previous findings^11^. Interestingly, the metabarcoding approach detected small proportions (~ 1–5%) of *S. aureus* reads 2 days and 1 month after decolonization in several noncarriers (Fig. 2B). This might reflect transient invasion of the nasal niche by *S. aureus* isolates, possibly facilitated by the disruption of the nasal microbiome induced by decolonization, as described in gut microbiota after antibiotic-induced perturbations^12^. This intermittent carriage is to be expected in the normal population.

### Disruption and recovery of the nasal microbiota after decolonization

**Figure 3:**
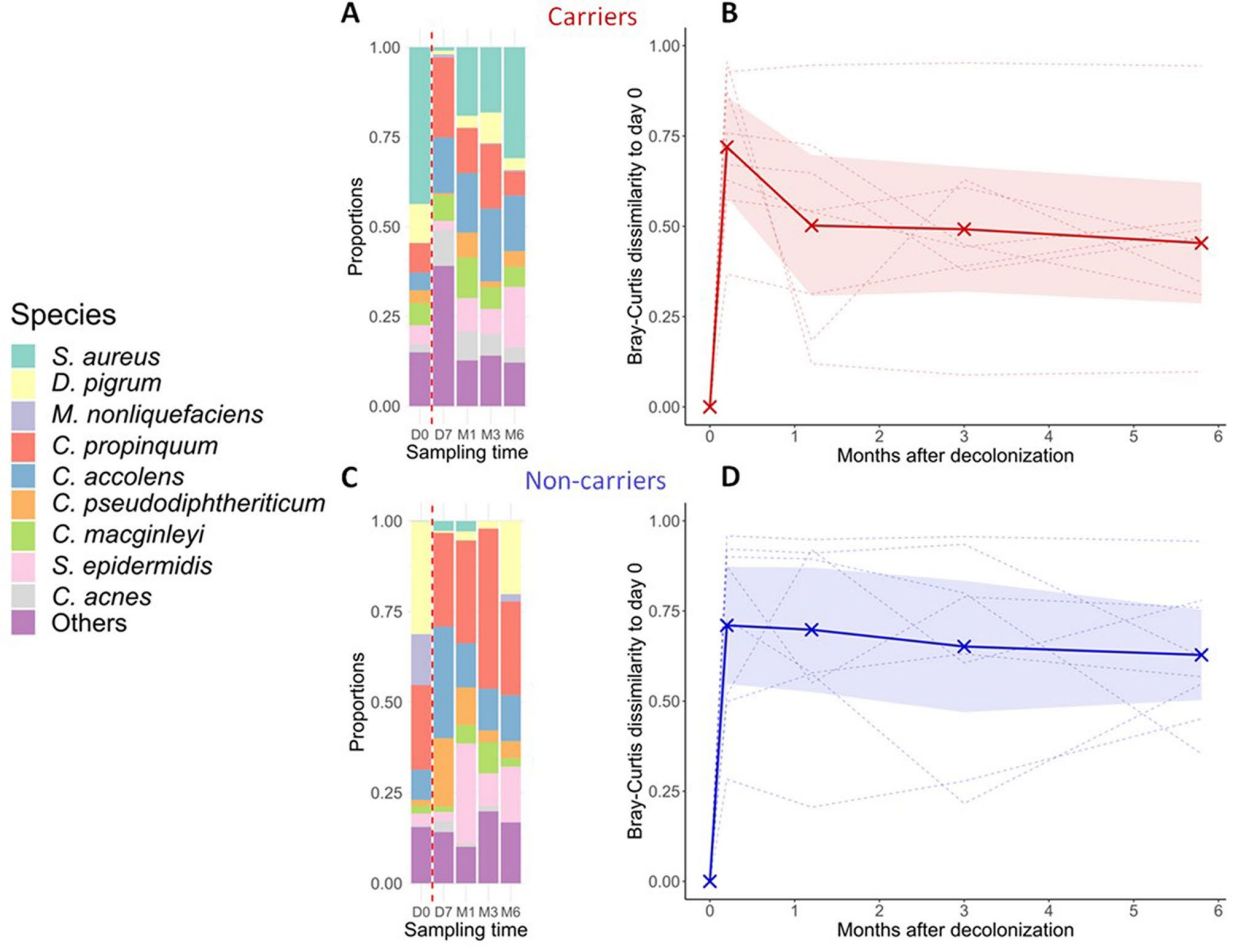
Evolution of the community structure of the nasal microbiota before and after mupirocin decolonization in *S. aureus* carriers and noncarriers. Shown are diversity bar plots of average species proportions (**A**,**C**) and the dissimilarity of these proportions (**B**,**D**) in each patient (dashed lines) and in average (solid lines; shaded area is the 95% confidence band of the mean). A high value for the Bray–Curtis dissimilarity indicates large difference in community structure relative to the initial state of the microbiota before decolonization. Nasal samples were taken immediately before decolonization (D0) and after 7 days (D7) and 1 (M1), 3 (M3), and 6 (M6) months in 8 *S. aureus* carriers (**A**,**B**) and noncarriers (**C**,**D**). Bacteria full names are, in order: *Staphylococcus aureus*,* Dolosigranulum pigrum*,* Moraxella nonliquefaciens*,* Corynebacterium propinquum*,* Corynebacterium accolens*,* Corynebacterium pseudodiphtheriticum*,* Corynebacterium macginleyi*,* Staphylococcus epidermidis*,* Cutibacterium acnes.*

Before decolonization, nine dominant bacterial species in nasal microbiota, including *S. aureus*,* S. epidermidis*,* D. pigrum*,* Moraxella nonliquefaciens*,* C. acnes* and 4 *Corynebactaria* species were identified (Fig. 3A,C; see details for each participant in Supplementary Fig. 2). *D. pigrum,* a common taxon found in the anterior nares, was particularly abundant and prevalent in noncarriers. *C. propinquum* was present in both groups and was in average 15% more abundant in noncarriers. Mupirocin-sensitive species, including *S. aureus* and *S. epidermidis*, *D. pigrum* and *M. nonliquefaciens*, were virtually removed from the microbiota after decolonization, while mupirocin-resistant corynebacteria and *C. acnes* remained substantially abundant^13,14^. After decolonization, the average proportion of *C. pseudodiphteriticum* in noncarriers, but not carriers, increased tenfold after 7 days and the proportion of *S. epidermidis* increased tenfold after 1 month. At other time points, the average proportions of *C. pseudodiphteriticum* and *S. epidermidis* were comparable in carriers and noncarriers. In the 2 carriers and 4 noncarriers colonized with more than 10% of *D. pigrum* (Supplementary Fig. 2), 1 was recolonized with *D. pigrum* after 1 month, 2 after 3 months and all after 6 months. *M. nonliquefaciens*, which was observed in 2 noncarriers, recolonized only 1 participant after 6 months. The median time to recolonization with *D.pigrum* and *M.nonliquefaciens*, 2 major taxa present in noncarriers, was 6 months. In contrast, the median time to recolonization of *S. aureus* in carriers was 3 months. Microbiota profiles of individual participants are shown in Supplementary Fig. 2.

To provide a more synthetic assessment of decolonization-induced changes of the microbial community structure, we computed the Bray–Curtis dissimilarity of the species assemblage at each time point, relative to the initial D0 time point in the same patient (Fig. 3B,D). The average Bray–Curtis dissimilarity was maximal immediately after decolonization in both carriers and noncarriers, denoting the most perturbed state of the microbiota. Strikingly, the dissimilarity decreased sharply in carriers but remained mostly stable in noncarriers, indicating that the microbiota of carriers (partially) reverted toward their initial state faster than in noncarriers, in line with faster recolonization by *S. aureus* compared to the dominant species found in noncarriers. After 6 months, the average dissimilarities from the initial state remained substantial (~ 0.5–0.7) both in carriers and noncarriers (Fig. 3B,D). Importantly, the evolution of population structure varied strongly across participants (see dashed lines in Fig. 3B,D), with microbiota recovery patterns ranging from fast recovery (dissimilarity < 0.2 after 1 month) to virtually no recovery (dissimilarity > 0.9 after 6 months) both in carriers and noncarriers.

## Discussion

In this longitudinal study of *S. aureus* carriers and noncarriers undergoing nasal mupirocin decolonization, we find that *S. aureus* recolonization in carriers occurred more rapidly than recolonization by the dominant species in noncarriers. These findings highlight the transient efficacy of the *S. aureus* decolonization procedure and the strong adaptation of *S. aureus* to the nasal niche.

Next to one case of failed decolonization, we observed frequent recolonization with *S. aureus* during the 6-month follow-up period. The time to recolonization ranged from 1 to 3 months, in line with previous observations in which the delay to recolonization ranged from 2 weeks to 6 months after mupirocin treatment^15^. In another longitudinal study collecting samples of 571 participants every 2 months for > 2 years, anti-staphylococcal antibiotics increased the rate of *S. aureus* acquisition within 4 months after treatment^16^, suggesting that microbiota disruption by antibiotics facilitates the invasion by *S. aureus*. In our study, 4 of 5 cases of recolonization eventually occurred with the same *spa-*type as isolated from the carrier initially. However, transient colonization with another *spa*-type was also demonstrated. This is in accordance with other studies showing longitudinal carriage of the same strain, with intermittent carriage of other strains as well^16–18^. While longitudinal studies suggest that loss and acquisition of *S. aureus* occur as natural events^16,18^, another reason for recolonization could be the lack of successful decolonization. Resistance to the decolonization treatment could facilitate recolonization. However, as Dutch national surveillance for resistance in *S. aureus* has shown low levels of mupirocin resistance (1%)^19^, it seems unlikely this would drive recolonization in our study participants. Recolonization from an untreated extra-nasal body site, such as the pharynx, or through household members is a more probable explanation.

Next to the loss of *S. aureus*, decolonization caused the immediate removal of *S. epidermidis*,* D. pigrum* and* M. nonliquefaciens* from the nose. In noncarriers, a trend towards a higher abundance of *C. propinquum* was observed, while an increase in *C. accolens* and *S. epidermidis* was shown in effectively decolonized carriers. Indeed, mupirocin treatment has been previously tied to an increase of the relative abundance of (unclassified) corynebacteria and *C. acnes*, along with a decrease of *S. epidermidis* abundance^20^. Together, these results imply a rearrangement of the nasal microbiota after decolonization treatment and the removal of mupirocin-susceptible species including *S. aureus*, allowing new taxa to invade the nasal niche.

Our study has limitations beyond its small sample size. To enhance study participation, we adopted a self-sampling strategy which allowed participants to send in samples through regular mail service. This method has been found appropriate for detection of *S. aureus* previously^21,22^. Nevertheless, delayed transport caused 20% of samples to be processed > 48 h after sampling. As only 3 of 27 delayed samples in carriers were culture-negative, the risk of false negative *S. aureus* cultures due to transport can be considered low. However, the impact of delay on metabarcoding approaches is unknown. Nevertheless, delayed transport had no effect on the overall recolonization results in this study.

Discrepancies were found between quantitative culture results and RNA metabarcoding regarding the presence of *S. aureus* in the post-decolonization samples. We defined recolonization based on a *S. aureus* positive culture (> 8 CFU/mL) after decolonization, consistent with our definition of *S. aureus* carriage. The varying nasal bacterial load, the intrinsic microbiota composition as well as potential influence of transport to the sequencing facility added to the multi-step RNA metabarcoding analyses are amongst the many factors explaining such differences with the culture results. Both methods agreed about recolonization status in three carriers only.

Overall, our findings highlight the sensitivity of the nasal bacterial community to mupirocin treatment and stress the fact that the decolonization target, namely *S. aureus*, re-enters the nasal niche comparably faster than the dominant species in noncarriers. This supports the current use of mupirocin as a short-term prevention procedure preceding an identified at-risk intervention, rather than a means of eliminating circulating *S. aureus* isolates.

## Methods

### Study population and study design

This is a prospective interventional cohort study of healthy *S. aureus* carriers and noncarriers in the Netherlands. All experiments were performed in accordance with the Dutch Medical Research Involving Human Subjects Act (WMO). The study protocol was approved by the local Medical Ethical Committee of the Erasmus University Medical Centre Rotterdam, The Netherlands (MEC-2018-091). Written informed consent was obtained for all participants. Participants were recruited through advertisements at Dutch universities and the research teams social networks. Exclusion criteria were age < 18 years, use of antibiotics, antiparasitics, antifungals or probiotics 3 months prior to recruitment, known allergy to components of the intervention treatment, pregnant and breastfeeding women, known chronic diseases affecting the immune system, severe chronic skin diseases, immunocompromised status, or use of immunosuppressant drugs.

After filling out an eligibility questionnaire, all volunteers were screened for *S. aureus* carriage as described previously^23^. *S. aureus* carriage was determined by quantitative culture of 2 weekly nasal swabs. Persistent *S. aureus* carriers were defined as 2 positive cultures with > 8 CFU/mL for each culture. Noncarriers were defined as 2 *S. aureus*-negative cultures. Intermittent *S. aureus* carriers were excluded from further participation in the study. Eligible volunteers were enrolled on a first-come, first-served basis.

Eligible participants were asked to fill out a questionnaire regarding risk factors for *S. aureus* acquisition. All participants received decolonization treatment. Decolonization consisted of mupirocin nasal ointment (2%, GlaxoSmithKline BV, Zeist, the Netherlands) twice daily and chlorhexidine gluconate cutaneous solution (4%w/v, Regent Medical Overseas Limited, Oldham, UK) once daily, both for 5 days.

Nasal samples were taken 1 day before decolonization (D0) and 2 days (D7), 1 month (M1), 3 months (M3) and 6 months (M6) after decolonization. All participants received a personal demonstration for nasal sampling by the executive researcher. Thereafter, all specimens were taken by the participants by inserting a swab (ESwab, 490CE.A, Copan Italia, Brescia, Italy) into one nostril and rotating 5 times, repeating this in the second nostril using the same swab. Swabs were collected in a container filled with 1 ml modified Liquid Amies, a collection and transport solution, and sent through regular mail service (non-temperature controlled) or deposited at the laboratory personally.

### *Staphylococcus aureus* quantitative culture

Quantitative *S. aureus* cultures were conducted to examine the dynamics of *S. aureus* carriage over the 6-month follow-up period after decolonization. Swab containers were vortexed for 20 s before plating. Serial dilutions of Amies medium were plated onto phenol mannitol salt agar (PHMA) and incubated for 2 days at 37 °C. Swabs were placed in phenol mannitol salt broth (PHMB) and incubated for 7 days at 37 °C for enrichment. *S. aureus* growth was confirmed by a latex agglutination test (Staph Plus Latex Kit, Diamondial, Vienna, Austria). Morphologically different *S. aureus* colonies were selected for *spa* typing and methicillin resistance screening using BBL CHROMagar MRSA II agar (BD, Breda, The Netherlands).

### *Spa* typing

Molecular typing of *S. aureus* isolates was performed to infer whether recolonization with *S. aureus* in decolonized carriers involved the same *spa-*type. Typing was limited to the last *S. aureus* positive culture moment and the last *S. aureus* positive culture moment after decolonization in recolonised carriers. *S. aureus* DNA lysates were prepared by boiling in 10 mM Tris–HCl, 1 mM disodium EDTA, pH 8.0 or extraction with the QIAamp DNA Mini Kit (QIAGEN, Venlo, The Netherlands) according to the manufacturer's instructions. Amplification of the *S. aureus* protein A (*spa*) repeat region was performed by PCR with 2 sets of primers. One set consisted of forward primer *spa-1113,* 5′-TAAAGACGATCCTTCGGTGAGC-3′ and reverse primer *spa-1514,* 5′-CAGCAGTAGTGCCGTTTGCTT-3′^24^. The other set consisted of forward primers *spa-F1,* 5′-AACAACGTAACGGCTTCATCC-3′ and *spa-F2* 5′-AGACGATCCTTCAGTGAGC-3′ and reverse primer *spa-R1* 5′-GCTTTTGCAATGTCATTTACTG-3′*.* Amplicons were purified with ExoSAP-IT (Applied Biosystems) according to the manufacturer’s instructions and sent for sequence analysis (Baseclear, Leiden, The Netherlands). Resulting sequences were analysed using BioNumerics v7.6 (Applied Maths NV, Sint-Martens-Latem, Belgium) and the *spa* types were assigned by use of the RidomStaphType database (Ridom GmbH, Würzburg, Germany).

### 16S ribosomal RNA sequencing of nasal microbiota

The impact of decolonization on the nasal microbiome and the recovery of the microbiome structure after decolonization were examined by means of 16S rRNA metabarcoding. Amies medium from each nasal swab container was stored at − 80 °C on the day of receipt at the study laboratory in Rotterdam, NL, then sent at − 80 °C to the microbiome analysis laboratory in Lyon, FR. To properly capture the impact of decolonization on the living microbiota, metabarcoding used RNA-based 16S ribosomal RNA (rRNA, which is preserved in living cells but quickly cleared after cell death or lysis) rather than the DNA coding sequence, as DNA can persist for prolonged time periods after cell death^25–28^. RNA was extracted using the Mag Bind^®^ Total RNA 96 Kit (Omega Bio-tek) tissue protocol from 150 µL of samples’ material. Cell lysis was performed using beads (Disruptor plate C plus—Omega Bio-tek) and proteinase K for 15 min at 2600 rpm, followed by 10 min at room temperature without agitation, and finished with a DNase I digestion of 20 min at room temperature. RNA was quantified using QuantiFluor RNA kit on Tecan Safire (TECAN). 10 ng total RNA was used for reverse transcription using FIREScript RT cDNA synthesis kit (Solis Biodyne) with random primers, then cDNA was purified with SPRIselect reagent (Beckman coulter) and quantified.

The rRNA V1–V3 region was PCR amplified using the 5× HOT BIOAmp^®^ BlendMaster Mix 12,5 mM MgCl 2 (Biofidal), 10× GC rich Enhancer (Biofidal) and BSA 20 mg/mL. The PCR reaction consisted of 30 cycles at 56 °C using the forward primer 27F, 5′-TCGTCGGCAGCGTCAGATGTGTATAAGAGACAG AGAGTTTGATCCTGGCTCAG-3′ and reverse primer 534R, 5′-GTCTCGTGGGCTCGGAGATGTGTATAAGAGACAGATTACCGCGGCTGCTGG-3′ in 25 µL of solution. PCR products were purified using SPRIselect beads (Beckman Coulter) in 20 µL nuclease-free water and quantified using QuantiFluor dsDNA (Promega). Samples were indexed with lllumina’s barcodes with the same PCR reagents during a 12 cycles PCR, then purified and quantified as previously mentioned. Samples were normalized and pooled, then sequenced using Illumina MiSeq V3 Flow Cell following the constructor’s recommendations for a 2 × 300 bp paired-end application. A mean of 130 k proofread reads per sample was obtained.

Experiment buffers were used as negative controls to detect contamination by out-of-sample bacterial RNA. RNA extraction was controlled using an in-house mix of live *Staphylococcus aureus* ATCC29213 and *Escherichia coli* ATCC25922 in equal proportions, allowing for assessing extraction bias in Gram-positive and -negative bacteria. PCR amplification bias was controlled using a commercial DNA mix of 8 bacterial species (ZymoBIOMICS™ Microbial Community DNA Standard).

### Bioinformatics and statistical analyses

Sequencing reads were quality checked and trimmed. Paired-ended read pairs were merged using BBMap version 38.49 (available at https://sourceforge.net/projects/bbmap/), with default options besides a minimum single size of 150 bp with an average Phred quality score higher than 10, and a total pair size of minimum 400 bp. PCR adapters were removed with cutadapt v.2.1 (Martin 2011) then dereplicated using vsearch v.2.12.0^29^ with the sizeout option. For species assignment, reads were aligned to sequences of NCBI blast 16S_ribosomal_RNA database (version date 03.12.2020) using Blastn v.2.11.0+^30,31^, keeping a maximum of 20 reference targets. Read counts per bacterial species were normalized to account for taxon-specific variations of the copy number of 16S rRNA genes using NCBI rrnDB-5.5 database based on the mean gene copy number in the taxon.

To optimize the resolution of sequencing read taxonomic assignment, we used in-house bioinformatic software publicly available at https://github.com/rasigadelab/taxonresolve. Briefly, when a read matches sequences from several species with identical alignment scores, taxonomic assignment pipelines typically output the higher taxonomic level such as the genus (e.g., *Staphylococcus* spp. when a read matches *S. aureus* and *S. epidermidis*). This loss of information can be problematic when species-level discrimination is important. To prevent losing species-level information, the *taxonresolve* software assigns reads with uncertain species to groups of species rather than to genera.

Bacterial species deemed present from contaminating sources such as kits reagents and found in negative controls, mostly from the *Bacillus* genera, were removed. A total of 1376 species or group of species were retained. The rarefaction curves corresponding to the sequencing effort to assess the species richness within samples are shown in Supplementary Fig. 3. Most samples reached a plateau after 40,000 sequences.

Given the small sample size compared to the number of variables and species considered in this study, no hypothesis testing was performed, and we provide a descriptive assessment of the results. In figures, 95% confidence intervals of the means were computed based on normal approximation, after log transformation for CFU/mL and log odds transformation for quantities restricted to the [0, 1] interval, such as proportions.

In microbial diversity analyses, we retained the 9 most prevalent bacterial species and pooled the other species into an ‘Others’ category. To assess the disruption and possible recovery of the microbiota, the divergence of sampled microbiota relative to the initial, pre-treatment microbiota (D0) was assessed using the Bray–Curtis dissimilarity at each sampling time point relative to the first sample of the same patient.

Software code of the analyses are available at https://github.com/rasigadelab/macotra-metabarcoding. Data are available at https://zenodo.org/record/6382657. Analyses and figures used R software v3.6.0^32^ with packages dplyr^33^, ggplot2^34^, vegan^35^, and MicrobiomAnalyst available at https://www.microbiomeanalyst.ca^36,37^.

## Supplementary Information

Supplementary Information.

## Data Availability

Datasets generated for this study are openly available in Zenodo at https://doi.org/10.5281/zenodo.6382657.
