# Supplementary material for: Nasal microbiome disruption and recovery after mupirocin treatment in Staphylococcus aureus carriers and noncarriers
Source: Sci Rep. 2022 Nov 17;12:19738. doi: 10.1038/s41598-022-21453-4 (PMC9671894; doi:10.1038/s41598-022-21453-4)
Supplement: Supplementary file 1 — Supplementary Information. [file 41598_2022_21453_MOESM1_ESM.pdf]

Nasal microbiome disruption and recovery after mupirocin treatment in *Staphylococcus aureus* carriers and noncarriers

Authors

Valérie O. Baede[1#], Anaïs Barray [2,3#], Mehri Tavakol[1], Gérard Lina [2,3], Margreet C. Vos [1¶], Jean-Philippe Rasigade[2,3¶*]

Author affiliations

1. Department of Medical Microbiology and Infectious Diseases, Erasmus MC University Medical Center Rotterdam, Rotterdam, the Netherlands
2. CIRI, Centre International de Recherche en Infectiologie, Inserm U1111, Université Lyon 1, Ecole Normale Supérieure de Lyon, Lyon, France
3. Centre National de Référence des Staphylocoques, Institut des Agents infectieux, Hôpital de la Croix Rousse, Hospices Civils de Lyon, Lyon, France

* Corresponding author

# Contributed equally

¶ Contributed equally

Supplementary information file

Members of the MACOTRA Study Group:

Valérie O. Baede [1], Anaïs Barray [2,3], Sake J. de Vlas [4], Anneke S. de Vos [4], Arya Gupta [5], Antoni P. A. Hendrickx [6], Gwenan M. Knight [7], Mirjam E. E. Kretzschmar [6,8], Gérard Lina [2,3], Jodi A. Lindsay [5], Jean-Philippe Rasigade [2,3], Leo Schouls [6], Mehri Tavakol [1], Margreet C. Vos [1], Willem J. B. van Wamel [1], Adam A. Witney [5]

Affiliations

1. Department of Medical Microbiology and Infectious Diseases, Erasmus MC University Medical Center Rotterdam, Rotterdam, the Netherlands
2. CIRI, Centre International de Recherche en Infectiologie, Inserm U1111, Université Lyon 1, Ecole Normale Supérieure de Lyon, Lyon, France
3. Centre National de Référence des Staphylocoques, Institut des Agent infectieux, Hôpital de la Croix Rousse, Hospices Civils de Lyon, Lyon, France
4. Department of Public Health, Erasmus MC University Medical Center Rotterdam, Rotterdam, the Netherlands
5. Institute for Infection and Immunity, St George's, University of London, London, United Kingdom
6. Center for Infectious Disease Control, National Institute for Public Health and the Environment (RIVM), Bilthoven, The Netherlands
7. Centre for Mathematical Modelling of Infectious Diseases, Infectious Disease Epidemiology, London School of Hygiene and Tropical Medicine, London, United Kingdom
8. Julius Center for Health Sciences and Primary Care, University Medical Center Utrecht, Utrecht University, Utrecht, The Netherlands

Supplementary table 1. Risk factors for *S. aureus* acquisition

		Cohorts	
		Noncarrier	Carrier
Total		8	8
Hormonal contraception		1	4
Dairy consumption			
	Daily	6	6
	Weekly	1	1
	Monthly	0	1
Disinfectants			
	Daily	3	1
	Weekly	2	1
Smoking			
	Daily	1	0
Alcohol consumption			
	Daily	1	2
	Weekly	2	2
	Monthly	2	3
	< 1x per month	2	1
Nose picking			
	Daily	5	2
	Weekly	2	5
	Monthly		1
	< 1x per month	1	
Household size			
	1		1
	2	5	5
	3	3	1
	4		1
Team sport or communal gym			
	Weekly	2	2
	Monthly		1
Sauna visits			
	< 1x per month	1	3
Healthcare or beauty practice			
	< 1x per month	1	2
Public transport			
	Daily	1	4
	Weekly	0	3
	Monthly	1	0
	< 1x per month	3	1

Supplementary Figure 1. *S. aureus* culture dynamics in carriers and noncarriers.

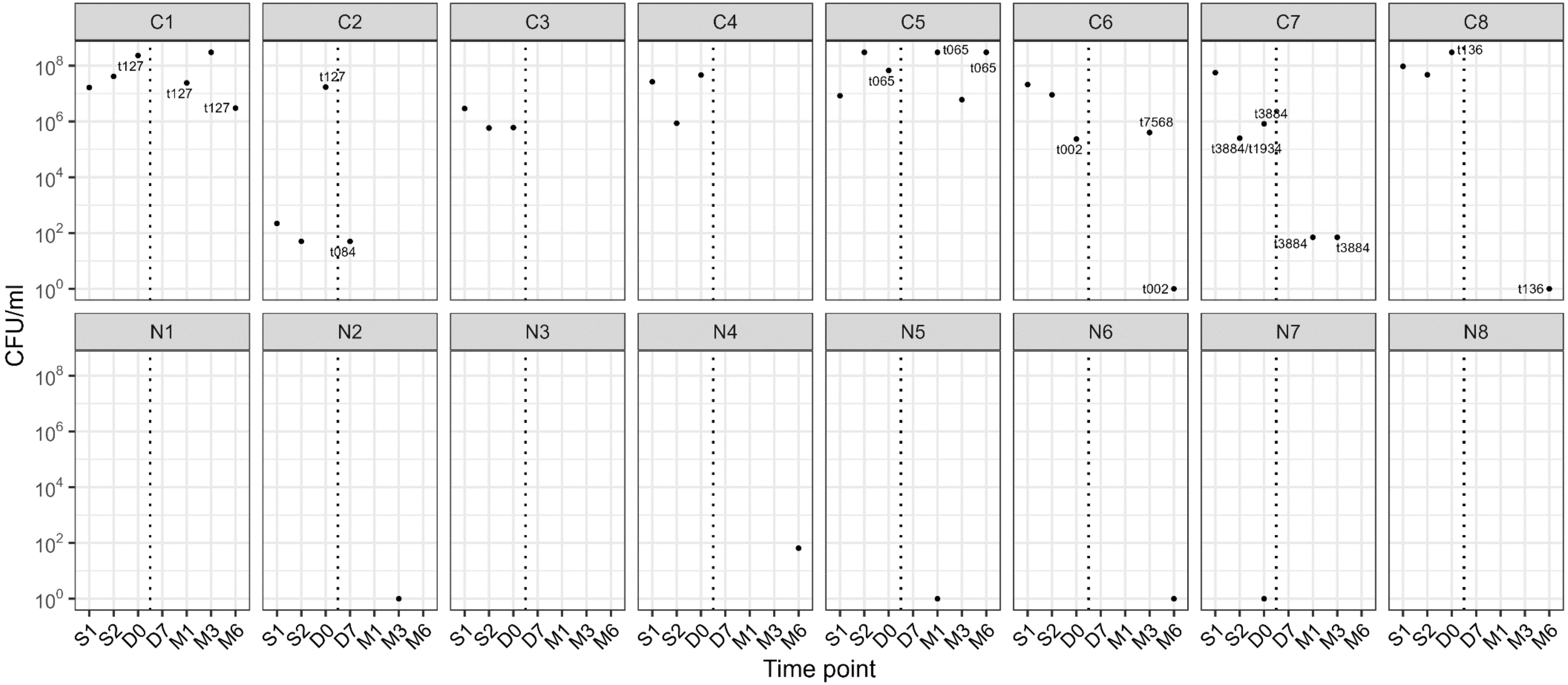

Sampling took place at screening time points S1 and S2. During the study phase, nasal samples were taken 1 day pre-treatment (D0) and 2 days (D7), 1 month (M1), 3 months (M3) and 6 months (M6) post-treatment. *S. aureus*-positive cultures are depicted as black dots, with bacterial load given on the y-axis. The vertical dotted line shows time of treatment. Spa-typing results are given as dot labels. Recolonization was defined as a *S. aureus* positive culture (>8 CFU/ml) post-decolonization

Supplementary Figure 2. Nasal microbiota of study participants in time.

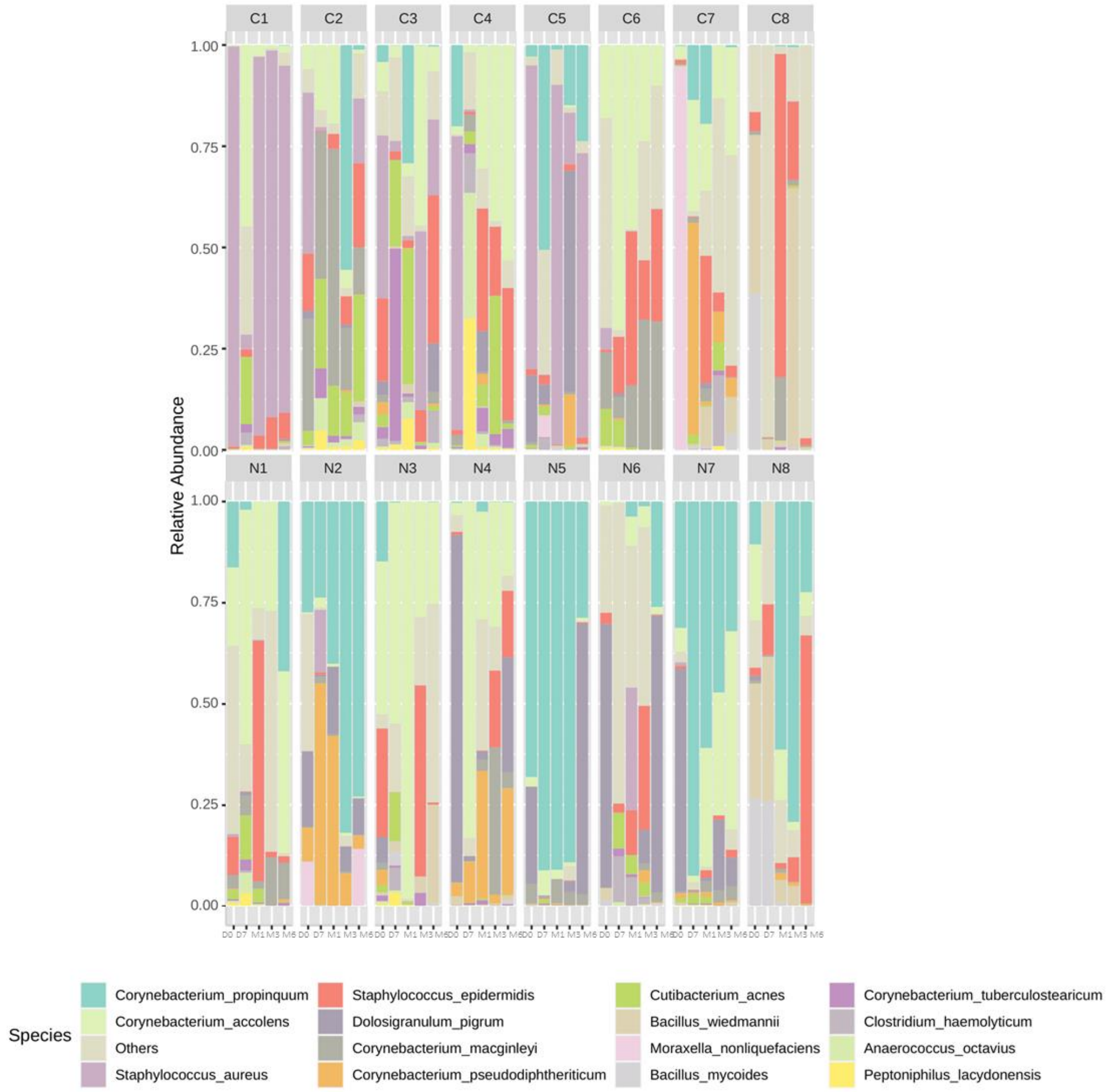

Shown are the detailed species proportions in nasal samples from 8 *S. aureus* carriers (top panel) and noncarriers (bottom panel), inferred through 16S rRNA metabarcoding. Samples were taken immediately before decolonization (D0) and after 7 days (D7) and 1 (M1), 3 (M3), and 6 (M6) months.

Supplementary Figure 3. Rarefaction plot

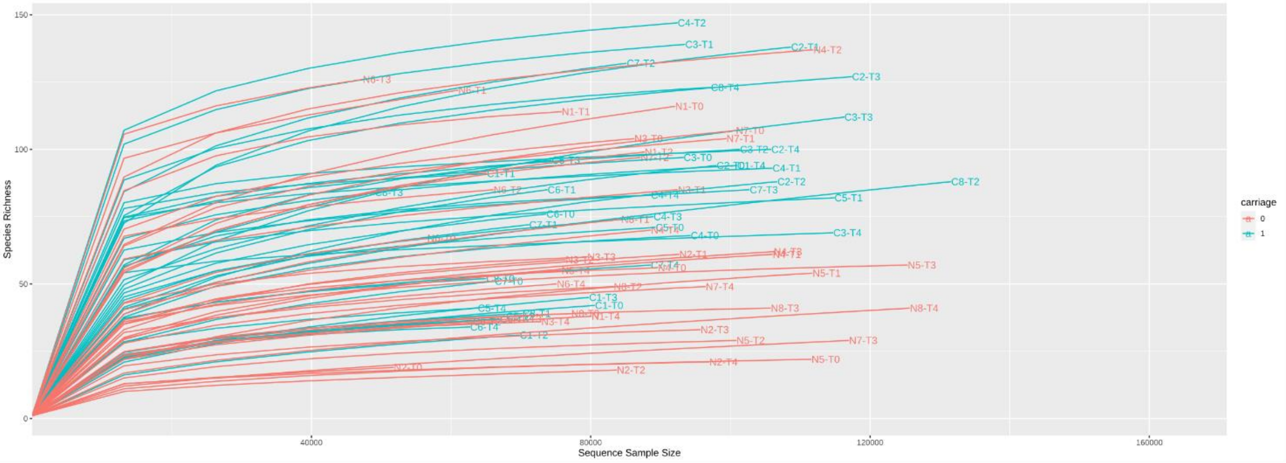

Rarefaction curves showing the number of sequences per sample and the belonging species richness uncovered ; carriers in red, noncarriers in blue. Curves reaching a plateau mean that a deeper sequencing would not result in significant species richness increase.
